# The Curve of Learning With and Without Instructions

**DOI:** 10.5334/joc.373

**Published:** 2024-06-03

**Authors:** Leendert van Maanen, Yuyao Zhang, Maarten De Schryver, Baptist Liefooghe

**Affiliations:** 1 Utrecht University, NL; 2 Ghent University, BE

**Keywords:** Decision making, Learning, Mathematical modelling

## Abstract

In skill acquisition, instructing individuals the stimulus-response mappings indicating how to perform and act, yields better performance. Additionally, performance is helped by repeated practice. Whether providing instructions and repeated practice interact to achieve optimal performance remains debated. This paper addresses that question by analyzing the learning curves of individuals learning stimulus-response mappings of varying complexity. We particularly focus on the question whether instructions lead to improved performance in the longer run. Via evidence accumulation modeling, we find no evidence for this assertion. Instructions seem to provide individuals with a head start, leading to better initial performance in the early stages of learning, without long-lasting effects on behavior. We discuss the results in light of related studies that do report long-lasting effects of instructions, and propose that the complexity of a skill determines whether long-lasting benefits of initial instructions exist.

Instructions play a beneficial role in the acquisition of new skills. Handling a new smartphone or camera without instruction is not only time consuming and error-prone, but also incurs unnecessary costs through trial-and-error learning. These costs can be easily bypassed by – even minimal – instruction of the condition-action rules or stimulus-response (SR) mappings that indicate how to handle different aspects of such device. Whereas previous research has focused on the effect of instructions at early stages of a task, the present study explores whether instructions also have a more long-lasting effect on task performance.

Previous research suggests that the role of instructions is mainly situated at the early stages of learning a new action or task, which Chein and Schneider (2012) referred to as the *formation phase* of learning. The formation phase reflects the phase of learning when new routines are established. In this early stage, learning on the basis of instructions presumably starts with the translation of linguistic information into a mental representation of a task (task model, Brass et al., 2017). This involves compiling a number of verbally instructed SR mappings in an action-oriented format when a task is simple (Hartstra et al., 2012; Ruge & Wolfensteller, 2010). However, with more complex tasks, the instructions also need to be structured hierarchically (Bhandari & Duncan, 2014; Duncan et al., 1996, 2008; Verbruggen et al., 2018). A task model is represented in activated long-term memory (Oberauer, 2009). In order to implement the instructed mappings, the relevant parts of the task model are activated further, such that they can lead to an almost reflexive response when triggered (Meiran et al., 2012, 2017). Once an instruction is actually performed, more temporarily stable traces are stored in long-term memory (Cohen-Kdoshay & Meiran, 2007, 2009; Liefooghe et al., 2012). The *controlled-execution phase* of the task is now possible (Chein en Schneider, 2012). When more practice is possible and stimuli are responded to repeatedly, more traces are formed, facilitating the emergence of skilled behavior through the automatic retrieval of these traces (Chein & Schneider, 2012; Logan, 1988, 1990). The *automatic-execution phase* is now achieved (Chein & Schneider, 2012).

The role of instructions in the controlled and automatic-execution phase seems futile. For instance, Schmidt, Liefooghe, and De Houwer (2020) proposed that early representations of instructions decay during the course of a task and are replaced by new traces formed on the basis of actual practice. However, a number of findings suggest that the effect of instructions may be more long-lasting than the formation phase. Abrahamse, Braem, De Houwer and Liefooghe (2022) consistently observed longer-term automatic effects of irrelevant but never executed instructions (see also Pfeuffer et al., 2017; Wenke et al., 2009). Such a finding is in line with research on prospective memory, which indicated that instructed but unexecuted intentions still bias behavior at later stages, even when they are irrelevant (Bugg & Scullin, 2013). Furthermore, Popp et al., (2020) observed that the initial chunking instructions in a discrete sequence-production task can influence performance even after several days of practice. Similarly, Rastle and colleagues (2021) demonstrated that instructions presented at the start of the formation phase can by-pass the effect of several hours of trial-and-error learning when acquiring a new language.

While models on instruction implementation mainly focus on performance of the very first trial following instruction encoding (Brass et al., 2017; Meiran et al., 2017), the impact of instructions on performance during the controlled and automatic execution phases of learning has not been adequately addressed. Here, we further document the more long-lasting effects of instructions by using learning curves (e.g., Newell & Rosenbloom, 1981). Learning curves show a non-linear improvement of performance, such as the decrease in reaction times as a function of practice. Learning curves were originally formalized by power functions (e.g., Newell & Rosenbloom, 1981). This function has been linked to cognitive models of learning, such as, ACT-R (Anderson & Milson, 1989; Anderson & Schooler, 1991), the component power laws model (Rickard, 1997), network models (Cohen et al., 1990), instance theories (Logan, 1988, 1990), or the chunking model (Rosenbloom & Newell, 1987). However, a substantial body of more recent evidence indicates that an exponential function offers a better fit of an individual learning episode and that the power function of learning is based on the distortion of aggregating across multiple learning episodes (Heathcote et al., 2000; see also Brown & Heathcote, 2003).

In an exponential function, the decrease in reaction times (RT) over practice is formalized in the following way (Heathcote et al., 2000):


\[
RT = R{T_0} + \;B{e^{ - \alpha R}}
\]

*RT*_0_ is the asymptote or minimal RT that is obtained after learning, i.e., in the automatic phase. *R* is the number of repetitions, *B* is the difference between initial performance and asymptotic performance, and *α* is the learning rate parameter, representing the speed by which *RT*_0_ is reached. The learning rate remains constant over practice in an exponential function. In contrast, in a power function, the learning rate decreases over practice. Initial models predicting a power function were thus challenged and had – or still need – to be accommodated with respect to this issue (see Heathcote et al., 2000 for a detailed discussion). Accordingly, we take abstraction from these different models and use the exponential function to test for long-lasting differences of instructions.

The central question of the present study is how instructions, which specify a completely new and arbitrary mapping between a stimulus and a response (e.g., “if Q, press left”) influence the performance improvement associated with the subsequent repeated execution of that mapping (the learning curve). To this end, the RT decrement over practice was compared between new SR mappings that were instructed and new SR mappings that were not instructed and had to be learned on the basis of feedback only (trial-and-error learning). At the center of this comparison are the three parameters of the exponential function (*RT*_0_, *B, α*), which specify different aspects of learning. Whether instructions are encoded into a task-set that enables prepared reflexes (Liefooghe et al., 2012; Meiran et al., 2015) or into SR episodes in memory that are automatically retrieved when target stimuli are presented (Abrahamse et al., 2022; Pfeuffer et al., 2017; Schmidt et al., 2020), instructions are expected to reduce the difference between initial performance and asymptotic performance (*B*). However, the effect of instructions on *α* and *RT*_0_ is less straightforward to predict. On the one hand, if we assume that instruction encoding leads to a task-set that represents SR mappings by configuring the cognitive system such that attention is biased towards relevant stimulus and response dimensions (e.g., Meiran, 2000; Vandierendonck et al., 2008, 2010), then this task-set should remain relatively activated, as long as the instructed SR mappings are relevant. The task-set established on the basis of instructions thus can speed up learning by facilitating the processing of relevant information. In contrast, when no instructions are provided, the task-set first needs to be constructed on the basis of trial-and-error learning. On the other hand, if we assume that instruction encoding leads to the formation of SR episodes in memory, more episodes are present following instruction encoding. This extra accumulation of episodes can also speed up performance beyond the first trial. Finally, learning without instructions leads to more errors. Such ‘noise’ may in turn hamper learning (see Ruge et al., 2018 for a similar point).

Taken together, existing accounts can be accommodated to predict that instruction encoding leads to longer lasting effects that go beyond the first-trial performance. As such, the learning rate could be increased, and asymptotic performance reached more quickly. Based on previous findings suggesting the presence of long-term effects of instructions (Abrahamse et al., 2022; Pfeuffer et al., 2017), improved learning may also result in improved asymptotic performance (*RT*_0_). At the same time, we need to be cautious with such a prediction as asymptotic performance may not be easily bypassed for simple choice-reaction time tasks. Consequently, we also considered the difficulty of the task to be learned and manipulated the number of SR mappings participants had to apply during a task, which could be 2, 4, 8, and even 16 SR-mappings. In the following, we formulated more specific predictions.

We predicted different ways in which instructing SR mappings at the start of task can influence learning in that task compared to a situation in which SR mappings are only learned via feedback (Figure 1). If instructions do not contribute to behavior at all, then learning should be identical to the blue curve in Figure 1, which represents feedback-only learning. If however, instructions do have long-lasting effects (Abrahamse et al., 2022; Bugg & Scullin, 2013; Pfeuffer et al., 2017; Popp et al., 2020; Wenke et al., 2009), then we expected a lower asymptotic performance, indexed by *RT*_0_ (the orange line in Figure 1). We referred to this hypothesis as *deeper encoding*, as it entails that instructions strengthen the memory traces in long-term memory, resulting in a continuing benefit on automatic performance. If, possibly in addition, instructions make the transfer to long-term memory easier, then we would expect *faster learning*, reflected by a higher learning rate *α* (yellow in Figure 1). Such a result would be consistent with the proposal by Abrahamse et al., (2022) that instructions initially help to transfer a skill to long-term memory. A third hypothesis that is also not precluded by Abrahamse et al., (2022), is that instructions help in the initial phase of learning a skill, but are not beneficial to the transfer of knowledge to long-term memory. In that sense, instructions would provide one with a *head start*, encoded by the initial performance level *B* being lower (green in Figure 1). This proposal would lead to a performance decrement during the formation and controlled execution phases of learning, but not during automatic execution.

**Figure 1:**
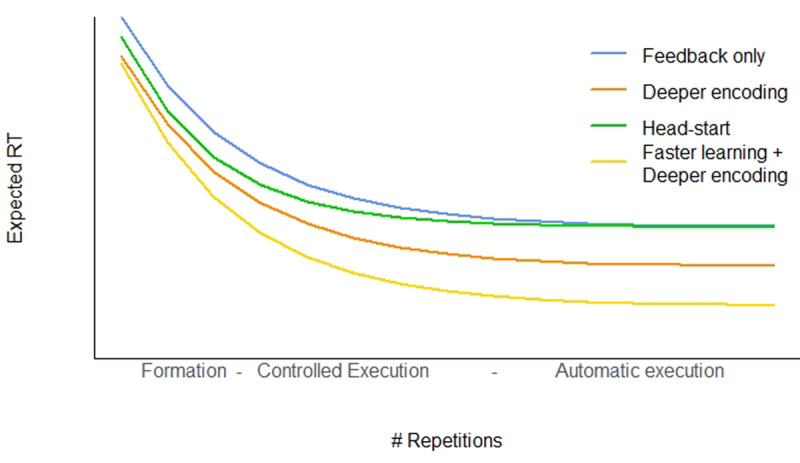
Different ways in which instructions could speed up responses in a stimulus-response mapping task. The blue curve represents baseline behavior; The orange, green, and yellow curves represent the predictions of different theoretical proposals (see text for details).

Foreshadowing our results, we found some evidence for an effect of instructions on *B*. Hence, instructions seem to provide a head start. The learning rate and asymptotic behavior were not influenced by instructions, suggesting that there are no long(er)-lasting benefits from learning by instruction. However, with respect to the asymptotic performance we reasoned that because the learning curve only considers RTs, we did not capture a possible speed-accuracy trade-off. To exclude this possibility, we additionally analyzed the data for which we observed asymptotic behavior using evidence accumulation modeling (EAM, Mulder et al., 2014; Ratcliff et al., 2016), in particular using the Diffusion Decision Model (DDM, Ratcliff & McKoon, 2008). This set of additional analyses again confirm that instructions do not improve asymptotic performance.

## Experiment 1

### Participants

A convenience sample of one hundred students at Ghent University participated for credit. Participants had normal or corrected-to-normal vision and were naive to the purpose of the experiment. Participants were randomly assigned to the Mapping conditions: 1:1 mapping (n = 20), 2:1 mapping (n = 20), and 4:1 mapping (n = 41), and 8:1 mapping (n = 19). The reason for the imbalance in the number of participants per cell comes from the fact that originally we planned for two experiments that differed in the way the SR mappings were initially presented (see the Design & Procedure section below). Since this manipulation did not affect behavior, we decided to collapse the two experiments (see also Appendix A for a statistical justification).

The study was approved by the local ethics committee at Ghent University, where the data was collected, under grant number BOF09/01M00209. All participants provided informed consent prior to participation. All data and analysis scripts of Experiment 1 are available on OSF: https://osf.io/q8sa2/.

### Design & Procedure

Participants performed blocks of a simple choice task, in which they had to respond to pictures depicting objects by either pressing a left (A) or a right key (P) on an AZERTY-keyboard. After an incorrect response, negative feedback was provided in the form of the message “fout!” (wrong), which was printed in red on the screen center. After a correct response, positive feedback was provided by printing the word “juist!” (correct) in green on the screen center. In half of the blocks the correct SR mappings were instructed at the beginning of each block (I+F blocks). In the remaining blocks, participants could only learn the SR mappings via feedback (F blocks). In the I+F blocks, SR mappings were provided by presenting the names of the stimulus objects either in a left or a right column on the screen. Objects whose name was presented in the left column were assigned to the left response-key and objects whose name was presented in the right column were assigned to the right response-key. In the F blocks, the message “READY?” appeared in the screen center. For half of the 4:1 mapping participants and all of the 8:1 mapping participants, object-names were also presented in the F blocks, in a single-column presented centrally on the screen, such that no response assignments could be inferred. This was done to eliminate a possible confound, namely that the object name were pre-exposed in the I+F blocks but not in the F blocks. Objects were depicted using the Snodgrass and Vanderwart (1980) pictures, their corresponding Dutch names were selected on the basis of naming norms (Severens et al., 2005). The number of objects (32, 16 assigned to I+F blocks and 16 assigned to F blocks) and the number of trials per objects (40) was the same in each condition. The four mapping conditions differed in the length and the number of I+F and F blocks (1:1 mapping: 8 F blocks, 8 I+F blocks; 2:1 mapping: 4 F blocks, 4 I+F blocks; 4:1 mapping: 2 F blocks and 2 I+F blocks; 8:1 mapping: 1 F and 1 I+F block). Accordingly, participants were always presented with 1280 trials.

Participants were tested in groups of two or three by means of personal computers with a 17-inch color monitor running Tscope (Stevens et al., 2006). Instructions were presented on screen and paraphrased. Depending on the mapping condition, participants performed a number of blocks, with a small break after each block. I+F and F blocks altered systematically. Block order was determined by the participants experimentation number: even numbered participants started with an I+F block. Depending on the mapping condition, SR mappings were presented for 20s (1:1 mapping), 40s (2:1 mapping), 1m20s (4:1 mapping) or 2m40s (8:1 mapping). The same time course was used for the F blocks. On each trial a picture depicting an object was presented on screen until a response was made or a maximum response time of 5000ms elapsed. Feedback messages were presented for 200ms. The inter-trial interval was set to 500ms.

### Fitting a learning curve

We estimated the optimal set of parameters by minimizing the sum of squared error between the observed RTs and the RTs predicted under the exponential model, using particle swarm optimization (Clerc, 2010). To understand which parameter best explains the observed RT differences between the experimental conditions, we performed model comparison between all models of the model hierarchy. That is, we systematically varied which parameters could differ between conditions, and fit all possible combinations of these. We started with the most complex model in which all three parameters were estimated separately for each condition (and individual), and iteratively applied simplicity constraints until the most constrained model was fit to the data, where all parameters were the same for both conditions (but separately for each individual). Then we compared the goodness-of-fit of all models while taking into account model complexity, approximated by the number of free parameters. The Bayesian Information Criterion (BIC, Schwarz, 1978) was computed according to the formula \[
BIC\; = k\log (n) + \;n\;{\mathrm{log}}(SSE{\mathrm{/}}n)
\] , with *n* being the number of observations per cell, and *k* the number of free parameters (see Van Maanen et al., 2019 for a similar approach). BIC was then used to compute BIC weights, which can be conceived as posterior probabilities that a specific model generated the data.

For the evaluation of the parameter estimates of the exponential learning curve, it is important to also include the uncertainty from the model comparison (Hinne et al., 2020; Hoeting et al., 1999). For this reason, we computed a weighted average of each parameter, where the weight is determined by the posterior probability of the model being the data-generating model (i.e., the BIC weight).

### Results

**Figure 2:**
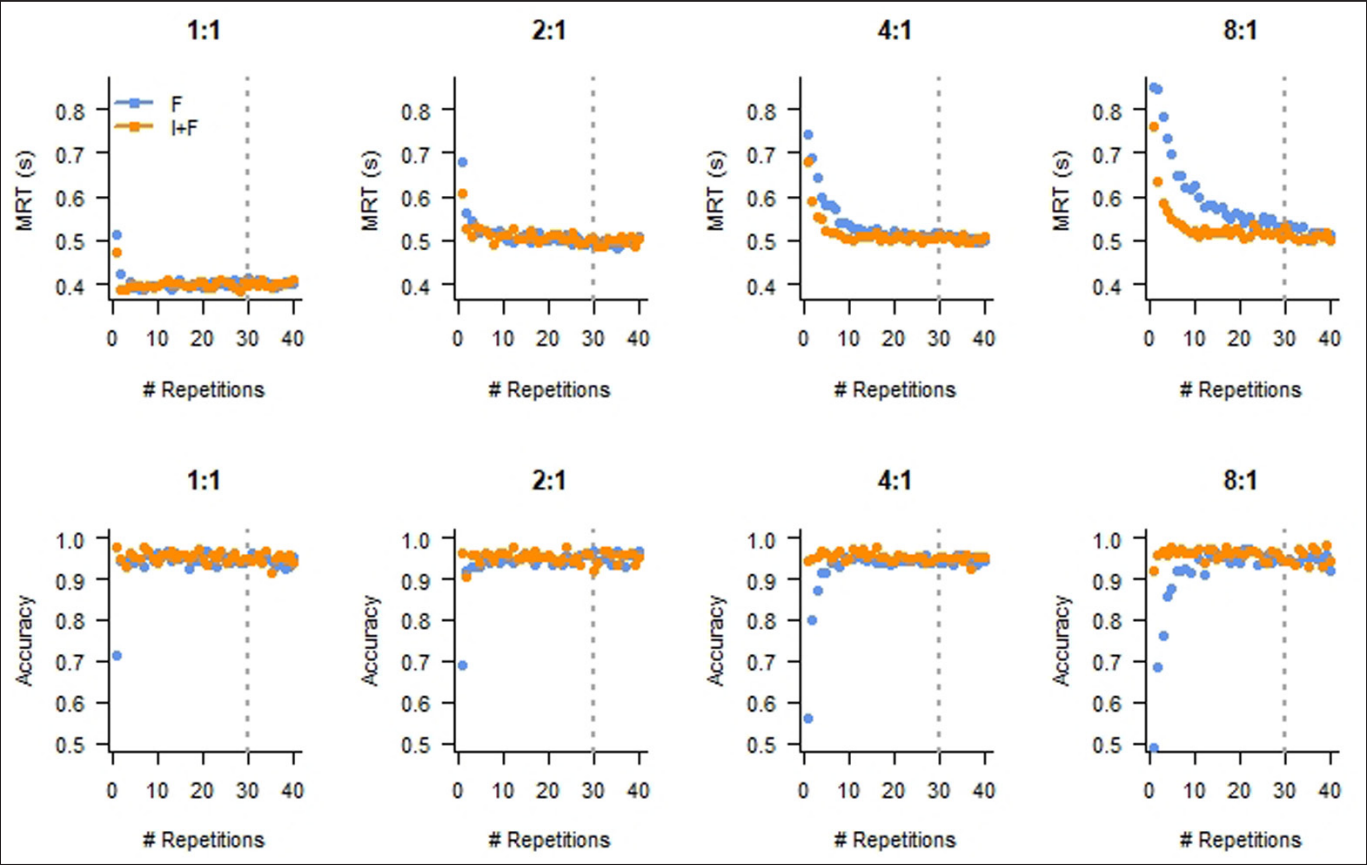
Mean response time (MRT, top row) and Accuracy (bottom row) as a function of Repetition reveal the expected performance improvement. Panels depict different Mapping conditions. F: Feedback only; I+F: Instruction and Feedback. The dashed lines indicate the repetition number after which we considered the data as showing asymptotic behavior.

We first considered RTs and accuracy rates. Figure 2 illustrates that over repetitions of the same SR mappings, participants speed up and make fewer errors. Moreover, it seems that this change in behavior is faster for the I+F condition, particularly for the more difficult mappings (4:1 and 8:1). To corroborate this observation, we analyzed the data with linear mixed-effects models on RT (Baayen et al., 2008). We included Instruction and Mapping as fixed effects, as well as whether a trial was from the first 10 repetitions of a SR pair, or the last 10 repetitions (we ignored the other trials). We treated participant as a random effect. The reference level was the Instruction condition (I+F) for the last 10 repetitions, in the 1:1 mapping.

This analysis revealed main effects on RT for Mapping (*ß*_2:1_ = 0.098, *t*(99.73) = 4.06, *p* < 0.001; *ß*_4:1_ = 0.10, *t*(99.73) = 4.93, *p* < 0.001; *ß*_8:1_ = 0.11, *t*(99.71) = 4.31, *p* < 0.001). Moreover, RTs were larger for the first 10 repetitions as compared to the last 10 repetitions (*ß*_first × 2:1_ = 0.026, *t*(62170) = 4.84, *p* < 0.001; *ß*_first × 4:1_ = 0.041, *t*(62170) = 8.70, *p* < 0.001; *ß*_first × 8:1_ = 0.066, *t*(62170) = 11.98, *p* < 0.001) and interacted with I+F, such that the differences between Mapping conditions were larger on the I+F blocks as compared to the F blocks (*ß*_first × 2:1 × I+F_ = 0.014, *t*(62170) = 1.76, *p* = 0.078; *ß*_first × 4:1 × I+F_ = 0.046, *t*(62170) = 6.94, *p* < 0.001; *ß*_first × 8:1 × I+F_ = 0.11, *t*(62170) = 13.70, *p* < 0.001). The only remaining significant effect on RT was an interaction between I+F and the 8:1 mapping (*ß*_8:1 × I+F_ = 0.015, *t*(62170) = 2.82, *p* = 0.0048).

For the accuracy, the interaction between Instruction and the first 10 repetitions was significant (compared to the last 10 repetitions, *ß*_first × F_ = –0.47, *z* = –3.03, *p* = 0.0025), as well as the three-way interactions with the more difficult mappings (*ß*_first × 4:1 × F_ = –0.48, *z* = –2.56, *p* = 0.010; *ß*_first × 8:1 × F_ = –0.92, *z* = –4.13, *p* < 0.001).

#### Learning curve

**Figure 3:**
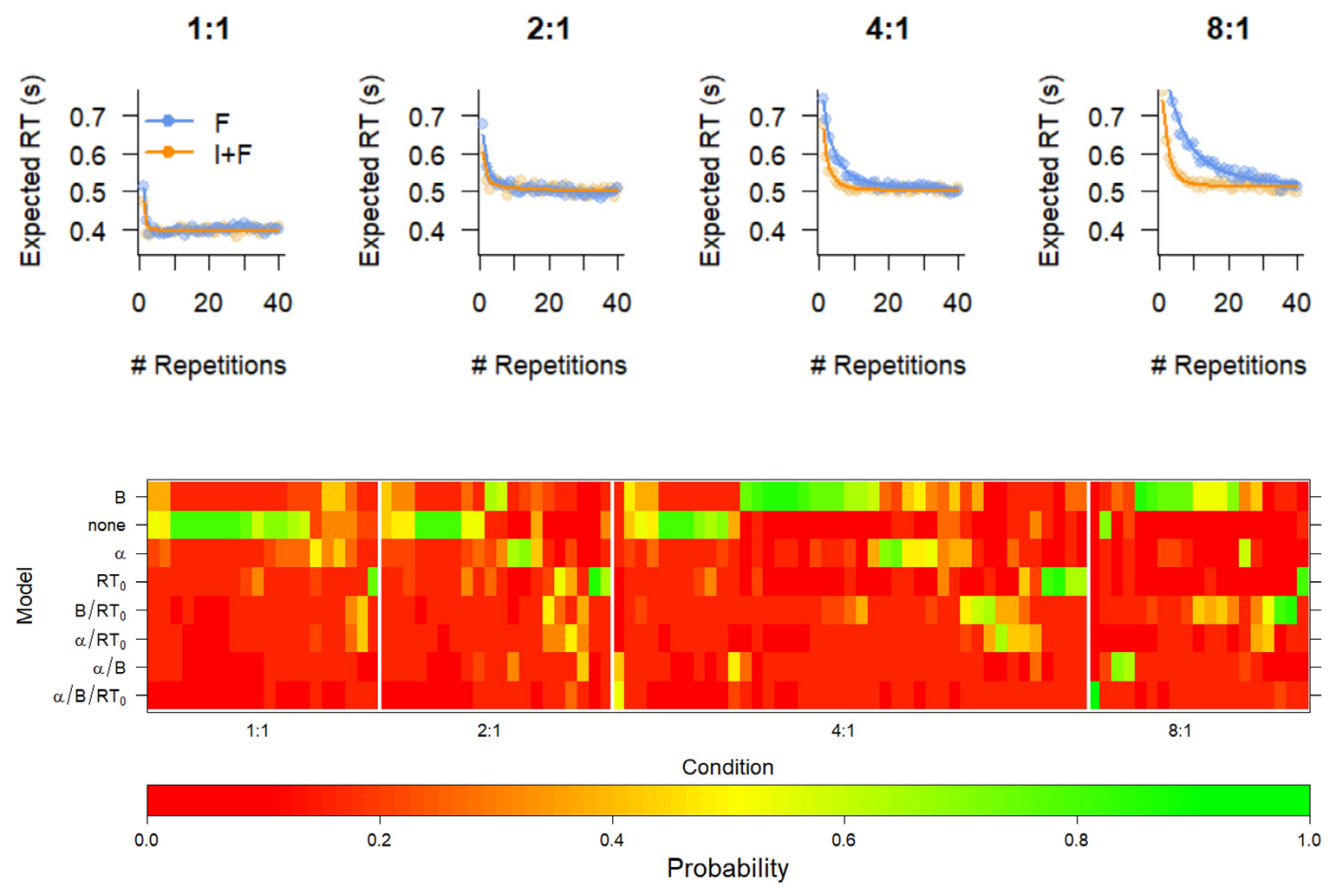
Fit of optimal model (top) and Model comparison (bottom) of various constraints on the exponential curve reveal that instructions boost the initial level of performance. Top. Mean response times (RT, points) and Expected RT (solid lines) of the B model. I+F: Instruction and feedback; F: feedback only. Bottom. BIC weights of all models for all participants. The models are indicated by which parameter was free to vary between F and I+F conditions. Participants are grouped according to a hierarchical clustering algorithm within the Mapping condition, for illustrative purposes. The colors represent the BIC weight.

Figure 3 (top) shows the averaged prediction of the best fitting model, and Figure 3 (bottom) shows BIC weights of all the models that we compared. Each column of the heatmap represents an individual participant, and each row represents a specific model. The models are ordered according to their overall best BIC, with the model that has the best balance between model complexity and goodness-of-fit on top. Participants are grouped according to a hierarchical clustering algorithm within the Mapping condition, for illustrative purposes. The colors represent the BIC weight, which expresses the probability that the data was generated according to a model with those specific constraints.

BIC model comparison reveals that the model with *RT*_0_ and *α* fixed across F and I+F conditions and *B* free to vary is overall preferred, although for a sizable subset of participants the simplest model without any differences between F and I+F conditions is preferred (the *none* model). Both these models support the hypothesis that Instruction-based learning has no lasting effect, since they both enforce the same parameter estimate for the asymptotic RT, independent of instruction.

The *none* model is clearly preferred for participants in the easier Mapping conditions; when comparing BIC weights for these two best performing models, we observed that the Bayes Factor (BF) of the *B* model over the *none* model increases over Mapping conditions, from clear support for the *none* model for the 1:1 mapping to clear support for the *B* model for the 8:1 model (BF_1:1_ = 0.20; BF_2:1_ = 0.46; BF_4:1_ = 1.90; BF_8:1_ = 8.68). This suggests that on the group level the learning of the simplest mappings is so fast that there is no additional benefit of instruction, while for the most complex mappings Instructions yield a boost to the initial learning.

**Figure 4:**
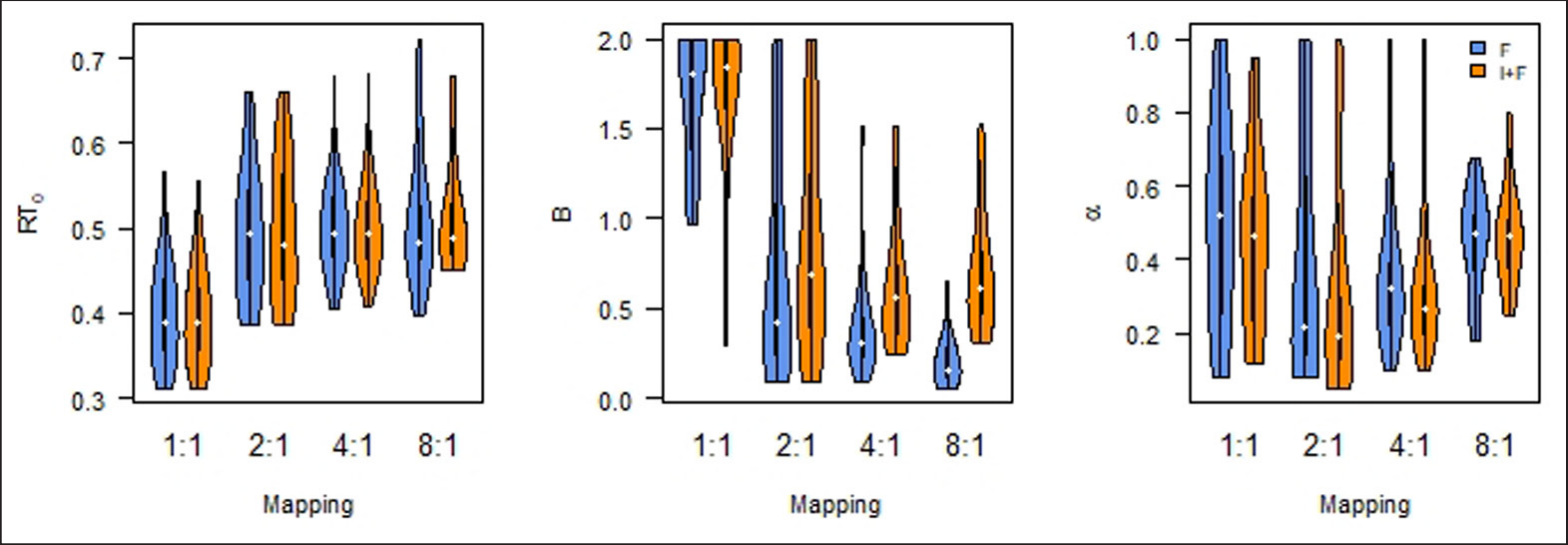
Asymptotic performance (*RT*_0_) differs by Mapping; Initial performance (B) differs by Instruction as well as Mapping. Learning rate (*α*) is not affected. F: Feedback only; I+F instruction and Feedback.

The same conclusion is reached when we analyzed the parameter estimates, as a weighted averaged over the models we compared (Figure 4). Bayesian ANOVA (Morey & Rouder, 2012; Rouder et al., 2012) reveals that *B* differs by Instruction (BF_10_ > 100), as well as by Mapping (BF_10_ > 100) and an interaction (BF_10_ > 100). Whereas *B* does not seem to differ between F and I+F for the 1:1 and 2:1 Mappings (although only limited support for the null hypothesis: BF_10_ = 0.36 and BF_10_ = 0.37, respectively), there is a difference for the for the 4:1 and 8:1 Mappings (both BFs > 100).

There is no clear effect of instruction on *α* (BF_10_ = 2.54), nor on mapping (BF_10_ = 2.25), or the interaction (BF_10_ = 0.32). Finally, there is evidence against an effect of instruction on the estimate of *RT*_0_ (Bayesian ANOVA, Rouder et al., 2012, BF01 = 6.57). At the same time, there is evidence for a difference in the estimates for *RT*_0_ related to the different Mapping conditions (BF_10_ > 100). This effect seems to be driven by a deviation of the 1:1 Mapping from the others. Pairwise Bayesian t-tests show Bayes Factors BF_10_ > 100 for comparisons with the 1:1 Mapping condition, and BF_10_ < 0.3 for the other comparisons. There is no evidence for an interaction (BF_10_ = 0.09).

## Interim Discussion

The results can be summarized as follows. On the one hand, instructions reduce the difference between initial and asymptotic performance in the learning process (i.e., *B*). On the other hand, instructions do not impact the learning rate itself (*α*) or asymptotic performance (*RT*_0_). Instructions thus do not seem to lead to long-lasting effects. However, learning curves on RT do not take into account the speed-accuracy trade-off that may appear within individuals and between conditions, potentially blurring true effects (Heitz, 2014; Wickelgren, 1977). Therefore, in the next section, we zoom in on the last 10 repetitions of each SR mapping and fit evidence accumulation models (EAM) to these data. This allows us to study potential differences between the F and I+F conditions in the joint distribution of RT and accuracy, thereby controlling for potential speed-accuracy trade-off effects.

## Diffusion Decision Model

The most-often used EAM is the Diffusion Decision Model (DDM, Ratcliff, 1978; Ratcliff & McKoon, 2008). This model assumes that in order to make a choice between two options, evidence representing the favored option is accumulated. A choice is made as soon as one of two boundaries that represent the two choice alternatives is reached. The average speed with which the evidence accrues is called the *drift rate* (*v*). If the drift rate has a positive value, evidence is accumulated for the option represented by the upper boundary; if the drift rate has a negative value, evidence is accumulated for the option represented by the lower boundary. If we assume that prior to the choice there is no preference for either option, the accumulation process starts exactly in the middle of the two boundaries. The distance between the boundaries then represents how much evidence is required to make a choice. This is the *boundary separation* (*a*), and the amount of evidence at the start of the trial is then *a/2*. Finally, the DDM also comprises time for stimulus identification and response execution, that adds up with the decision time to the total response time. These additional time components are referred to as the *non-decision time* (*t*_0_). In the full diffusion model applied here, all these mechanisms consist of a mean parameter value and a parameter representing between-trial variability around that mean.

### Methods

The model was fit on the RT distributions of correct and incorrect responses. That is, we collapsed over left and right responses, and over all possible stimuli. Following Ratcliff and Tuerlinckx (2002), we assumed 5% RT contaminants, which we modeled by a uniform distribution ranging from the fastest to the slowest RT per participant and condition. Because our primary focus is on the potential lasting effects of Instruction-based learning, we only included the last 10 repetitions of each SR mapping. Based on the overall RTs, it seemed that most individuals had learned the SR mappings in each condition after 30 repetitions. The final 10 repetitions thus serve as an estimate of the automatic execution phase, with reasonably stable behavior. The DDM as we apply it here assumes that all observations are independent, which makes stable behavior a precondition for reliably interpreting the model fit and the parameters. Moreover, analyzing the full learning curve using DDM revealed issues with model identifiability (see also Van Maanen & Miletić, 2021), or interpretability issues in itself (Miletić et al., 2020). Our approach of fitting the standard DDM to the final set of repetitions seems to provide the best trade-off between rigor and interpretability. We fit the model to the data of individual participants by minimizing the negative summed log likelihood of the data (-SLL) of all trials under a set of parameters, using Particle Swarm optimization (Clerc, 2010) with default settings.

As with the learning curves, we performed model comparison to identify differences between experimental conditions. We again started with fitting the full model, after which we constrained the three main parameters (*v, a, t*_0_) over conditions, together with their between-trial variability counterparts^1^ that we included as nuisance parameters. Then, we constrained combinations of those parameters, until we finished with the simplest model. We again computed BIC and BIC weights (Wagenmakers & Farrell, 2004), but because the models were fit by minimizing -SLL, the following formula was used: \[
BIC = k{\mathrm{log}}(n) - 2SLL
\] , with *n* the number of observations per cell, and *k* the number of free parameters. For the evaluation of the parameter estimates of the DDM, we computed BIC weighted parameters (Hinne et al., 2020; Hoeting et al., 1999).

### Results

**Figure 5:**
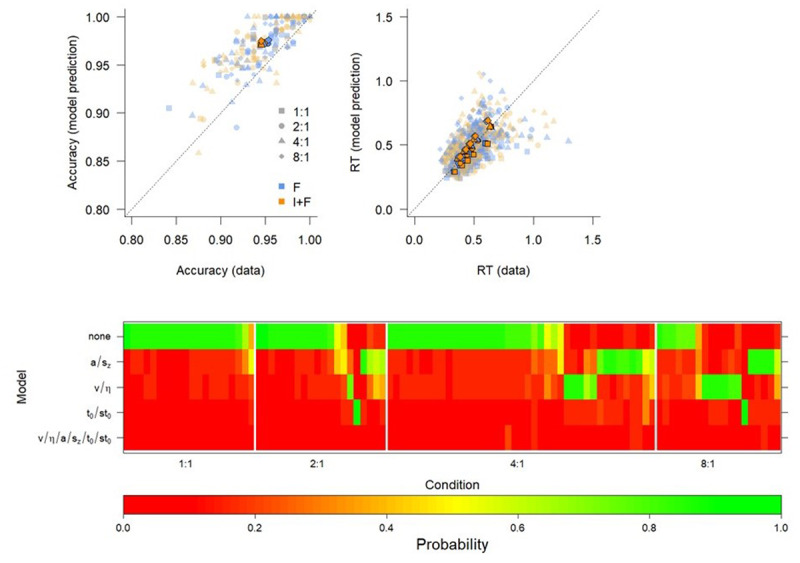
Fit of optimal model (Top panel) and model comparison (Bottom panel) of various constraints on the Diffusion Decision Model reveal that there are no lasting effects of instruction. Top. QQ-plots of model fits of the none model. Individual data points refer to individuals, thick shapes indicate the mean of the 0.1, 0.3, 0.5, 0.7, and 0.9 quantile RTs (cf. Miletić & Van Maanen, 2019). I+F: Instruction and feedback; F: feedback only. Bottom. BIC weights of all models for all participants. The models are indicated by which parameter was free to vary between F and I+F conditions. Participants are grouped according to a hierarchical clustering algorithm within the Mapping condition, for illustrative purposes. The colors represent the BIC weight.

Figure 5 (top) shows the averaged prediction of the overall best fitting model, with Figure 5 (bottom) showing the BIC weights of all DDM specifications that we compared. It is clear that for all mapping conditions except 8:1, the model that assumes no difference between conditions is preferred. For the 1:1 mapping condition, 19 out of 20 participants are best described by this simplest model; for the 2:1 mapping condition, 13 out of 20 are best described by this model; and for the 4:1 mapping condition, 27 out of 41 are best described by this model. For the 8:1 mapping condition, the simplest model is also preferred for 6 out of 19 participants, but the majority of participants are best described by a model that assumes a difference between the F and I+F conditions (13 out of 19).

Overall, it seems that the model without any differences between conditions (the “none” model) is preferred, especially when it is clear that learning has reached asymptotic behavior.

**Figure 6:**
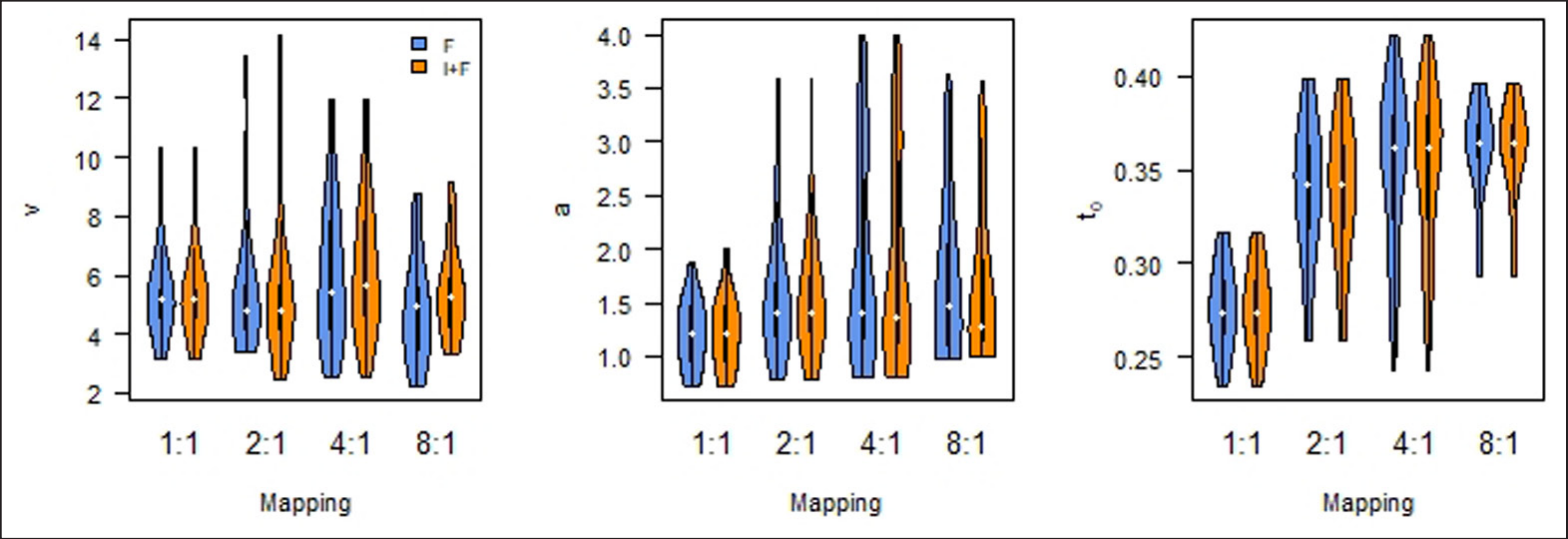
Diffusion Decision Model parameters by mapping. Left: drift rate; Middle: boundary separation; Right: non-decision time. F: Feedback only; I+F instruction and Feedback.

Bayesian ANOVA (Rouder et al., 2012) on the model-averaged parameters, with Instruction, Mapping, and their interaction as fixed effects and Participant as random-effect, reveals evidence in favor of an effect of instruction on drift rate (BF_10_ > 100), as well as an interaction effect (BF_10_ = 8.55, Figure 6). These effects seem to be driven by a higher drift rate for Instruction in the more difficult Mapping conditions. Post hoc Bayesian t-tests indeed support this, with weak evidence in favor of the absence of an effect for the 1:1 and 2:1 Mappings (BF_01_ = 2.68 and BF_01_ = 3.64, respectively), but strong evidence for the presence of an effect for the 4:1 and 8:1 Mappings (BF_10_ = 8.40 and BF_10_ = 7.81, respectively). On average, drift rates are higher in the Instruction condition (mean v_4:1_ = 6.1 and mean v_8:1_ = 5.4) than in the Feedback only condition (mean v_4:1_ = 5.9 and mean v_8:1_ = 4.9). These differences are small as compared to the average drift rates in the 1:1 and 2:1 mapping conditions (mean v_1:1_ = 5.3 and mean v_2:1_ = 5.4).

For the boundary separation, there is no evidence for an effect of instruction, nor mapping (BF_10_ = 0.69 and BF_10_ = 1.38, respectively). For the non-decision time, the Bayesian ANOVA model that included Instruction as a factor did not converge. This is because for almost all of the DDMs that best describe individual behavior, the non-decision time parameters did not vary by instruction. Thus, fitting an ANOVA model including this factor becomes meaningless. The ANOVA with only Mapping as a factor supports the observation that non-decision time differs as a function of mapping difficulty (BF_10_ > 100). Specifically, the 1:1 Mapping yields a lower non-decision time than all other mappings (post-hoc Bayesian t-tests, all BF_10_ > 100; mean t_0,1:1_ = 0.27; mean t_0,2:1_ = 0.34; mean t_0,4:1_ = 0.35; mean t_0,8:1_ = 0.36). However, the evidence for differences between the other mapping conditions is inconclusive (0.37 < BF_10_ < 1.65).

### Relationship between learning and stable behavior

We observed that participants, after repeated execution of the SR mappings, generally reached stable behavior that is comparable across F and I+F conditions. This was measured both by asymptotic response times and via the application of an evidence accumulation model (DDM) on the last 10 repetitions of each stimulus (i.e., the automatic execution phase). For the more complex SR mappings however, it appeared that some participants had not reached that level of optimal performance yet, despite evidence suggesting this was the case at the group level. Therefore, we reasoned that a difference between F and I+F conditions that was observed in the DDM parameters, may be the result of participants having not yet reached asymptotic behavior. To support this hypothesis, we studied the association between the stable behavior and the DDM.

**Figure 7:**
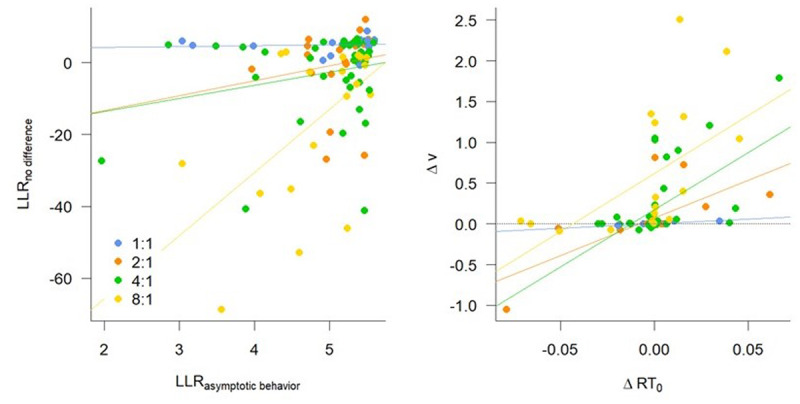
Participants that have not reached asymptotic behavior are best characterized by differences in drift rate and boundary separation. Left: A higher log likelihood ratio (LLR) in favor of reaching asymptotic behavior predicts a higher evidence ratio in favor of no difference between Diffusion Decision Model (DDM) parameters for the last 10 repetitions. Right: A difference in asymptotic RT (*RT*_0_) predicts a positive difference in drift rate (v).

We discovered evidence for a decrease in RT in the last 10 repetitions – evidence that individuals were still learning and have not yet reached the asymptotic RT – predicted the probability that there was a difference between F and I+F conditions in terms of the DDM analysis (Figure 7, Left panel). This was particularly the case for the 8:1 Mapping condition. Specifically, for each participant, we quantified the evidence for a decrease in RT as the log likelihood ratio of the slope of a linear regression of Repetition on RT being smaller than 0 (Dienes & Mclatchie, 2018), indicating a negative trend in RTs. These LLR_Slope_ predicted the log likelihood ratio (LLR_DDM_) of the DDM models that assume any difference between conditions versus the *none* model that assumes no difference, controlling for Mapping (Figure 7, Left panel, BF_10_ > 1000). This effect was primarily driven by individuals in the 8:1 Mapping condition: A model including the Repetition × Mapping interaction was 4.27 times more likely than the model without interaction, and the model that only included the 8:1 Mapping condition was 4.30 times more likely than the intercept-only model.

The differences in asymptotic RT between F and I+F conditions seem to be mainly associated with differences in drift rates (Figure 7, Right panel). We compared Bayesian regression models that – in addition to the Mapping – included the normalized difference between model-averaged asymptotic RTs for the conditions as predictors, and the normalized difference between model-averaged DDM parameters as dependent variables. The regression model with the *RT*_0_ difference as well as Mapping as predictors for the drift rate difference was over 10,000 times more likely than a model with Mapping only (over 1000 times more likely than a model including the *RT*_0_ difference but without Mapping, and ~27 times more likely than the full model including the interaction). This suggests that individuals who still benefit from instructions after 30 repetitions of the items, have a higher drift rate in the I+F condition then in the F condition (mean effect: 0.49, sd = 0.081).

## Experiment 2

The results of Experiment 1 suggested that instructing SR mappings mainly provides a head start when a task begins, but has no lasting effect on automatic task execution. Additional analyses showed that the difference in DDM parameters we observed on the final 10 trials could be explained by participants that did not reach asymptotic performance yet. To corroborate our suspicion that instructions do not seem to have a lasting influence on choice behavior after many repetitions, we conducted a follow-up experiment. In Experiment 2, participants had to perform the same task as in Experiment 1, with an 8:1 stimulus-to-response ratio. Importantly, the number of repetitions was increased to 60, to ensure that all participants indeed learned the mapping at the end of the experiment.

### Methods

Experiment 2 was ran online and hosted in Gorilla (Anwyl-Irvine et al., 2020). The task required participants to learn 16 SR mappings (that is, an 8:1 mapping). Twenty pictures were selected from the Snodgrass and Vanderwart (1980) database that corresponded to high frequent English object names consisting maximally of six letters and two syllables. Each picture was presented 60 times. In view of the online setting, the instruction manipulation was between subjects. Consequently, the experiment only lasted for a maximum of 30 minutes. Participants first received general instructions, with a small practice block of 12 trials to get familiarized with the task, including the encoding of the SR mappings or previewing the object names when no SR mappings were instructed. Next, the actual task began. In order to keep participants engaged in the online setting, a progress bar was added. Time parameters were the same as in the 8:1 mapping condition of Experiment 1. Feedback was now provided by presenting a green “✔” after a correct response and a red “✖” after an incorrect response.

We collected data of 140 participants (I+F condition: 69, *M_age_* = 22.56; *SD_age_* = 1.80; 35 Female, 33 Male, 1 undisclosed; F condition: 70, *M_age_* = 22.33; *SD_age_* = 1.71; 36 Female, 33 Male, 1 undisclosed). After initial screening of the data, we noticed that a sizeable subset of individuals did not engage in the task and therefore did not learn the SR mappings, as evidenced by their RT patterns. To exclude individuals that did not learn the task, we compared RTs of the first 200 trials with RTs of the final 200 trials. Participants that were on average *slower* on the last 200 trials than the first were excluded from further analyses (21 individuals). Additionally, participants that overall did not perform over 60% accuracy were also excluded (13 individuals). Finally, we excluded two individuals that used multiple browsers, as evidenced by the log files, two individuals that on average required more than 2 seconds per trial, and one individual that was on average faster than 150 ms. Overall, we retained 104 participants. The data and analysis scripts of Experiment 2 are available on OSF: https://osf.io/q8sa2/.

We performed similar analyses as for Experiment 1. That is, we first reported the results of linear mixed effects modeling. Then, we estimated learning curves for each individual in the same way as for Experiment 1, and then fit DDM to the stable behavior of the individuals. In contrast to Experiment 1, we now fit the DDM to the last 15 repetitions, increasing the number of observations to improve the parameter estimation. This change entails that we assumed that learning behavior has stabilized after 60–15 = 45 repetitions, up from 40–10 = 30 repetitions in Experiment 1.

Because the experiment used a between-participant design, we could not perform model comparisons in the same way as for Experiment 1. Instead, we only estimated the most complex models, and drew inferences from the estimated parameters, for both the learning curves and DDMs.

### Results

We fit a linear mixed-effects model on RT including the first and last 10 repetitions and Instruction as fixed effects, and participants as random effects to the RT data of Experiment 2. The reference level was the Instruction condition (I+F) for the last 10 repetitions. This analysis revealed that the first 10 repetitions were slower than the last 10 repetitions, indicating that participants did learn the task (*ß*_first_ = 0.264, *t*(295900) = 34.09, *p* < 0.0001). Moreover, there was a significant interaction between the first 10 Repetitions and the Instruction, such that RTs were higher for the first 10 repetitions in the Feedback only condition (*ß*_first × F_ = 0.089, *t*(296000) = 7.67, *p* < 0.001). This shows a successful operationalization of the task, and the initial effect of instruction. In contrast, there was no significant effect of Instruction for the last 10 repetitions (*ß*_F_ = 0.082, *t*(106) = 1.85, *p* = 0.067).

The accuracy results were in line with these findings. We fit generalized linear mixed effects models with a logistic link function and the same factors as for RT to the accuracy data of Experiment 2. Accuracy was lower in the first 10 repetitions than the last (*ß*_first_ = –0.17, *z* = –2.96, *p* = 0.0030). There was a significant interaction between the first 10 repetitions and instruction ((*ß*_first × F_ = –1.62, *z* = –20.19, *p* < 0.001), but no main effect of Instruction on the last 10 repetitions (*ß*_F_ = 0.0013, *z* = 0.009, *p* = 0.99).

#### Learning curve

**Figure 8:**
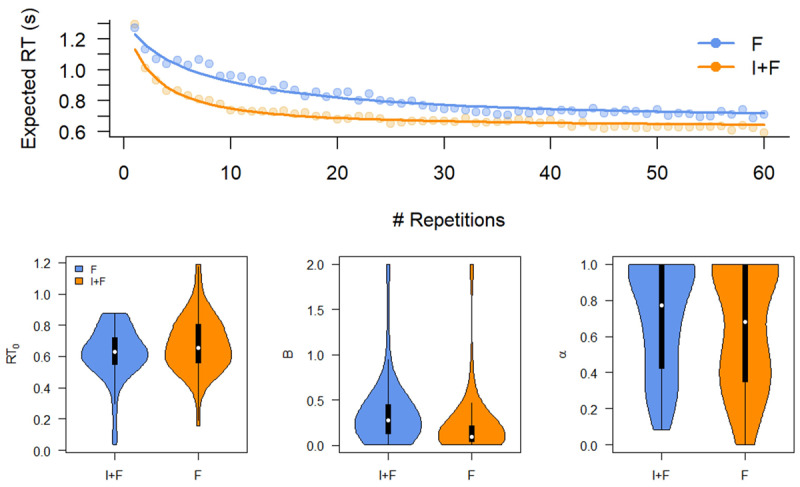
Model fits and parameter estimates for an exponential learning curve fit to the data of Experiment 2. Top. Mean response times (RT, points) and Expected RT (solid lines). Bottom. Asymptotic performance (*RT*_0_), initial performance (B), and learning rate (*α*) do not differ between conditions. F: Feedback only; I+F instruction and Feedback.

Figure 8 shows the results of fitting an exponential curve to the reaction times of Experiment 2. Although the optimal fit suggests that after 60 repetitions there is an RT difference between I+F and F conditions (Figure 8, top), this is not supported by the distribution of the parameter estimates (Figure 8, bottom). That is, we found Bayes factors in support of the null hypothesis, albeit that these were small and only provide anecdotal evidence for an absence of a difference (BF_01_ = 1.52; BF_01_ = 1.24; BF_01_ = 3.29, for *RT*_0_, B, and *α* respectively, in support of the null hypothesis).

#### Diffusion Decision Model

**Figure 9:**
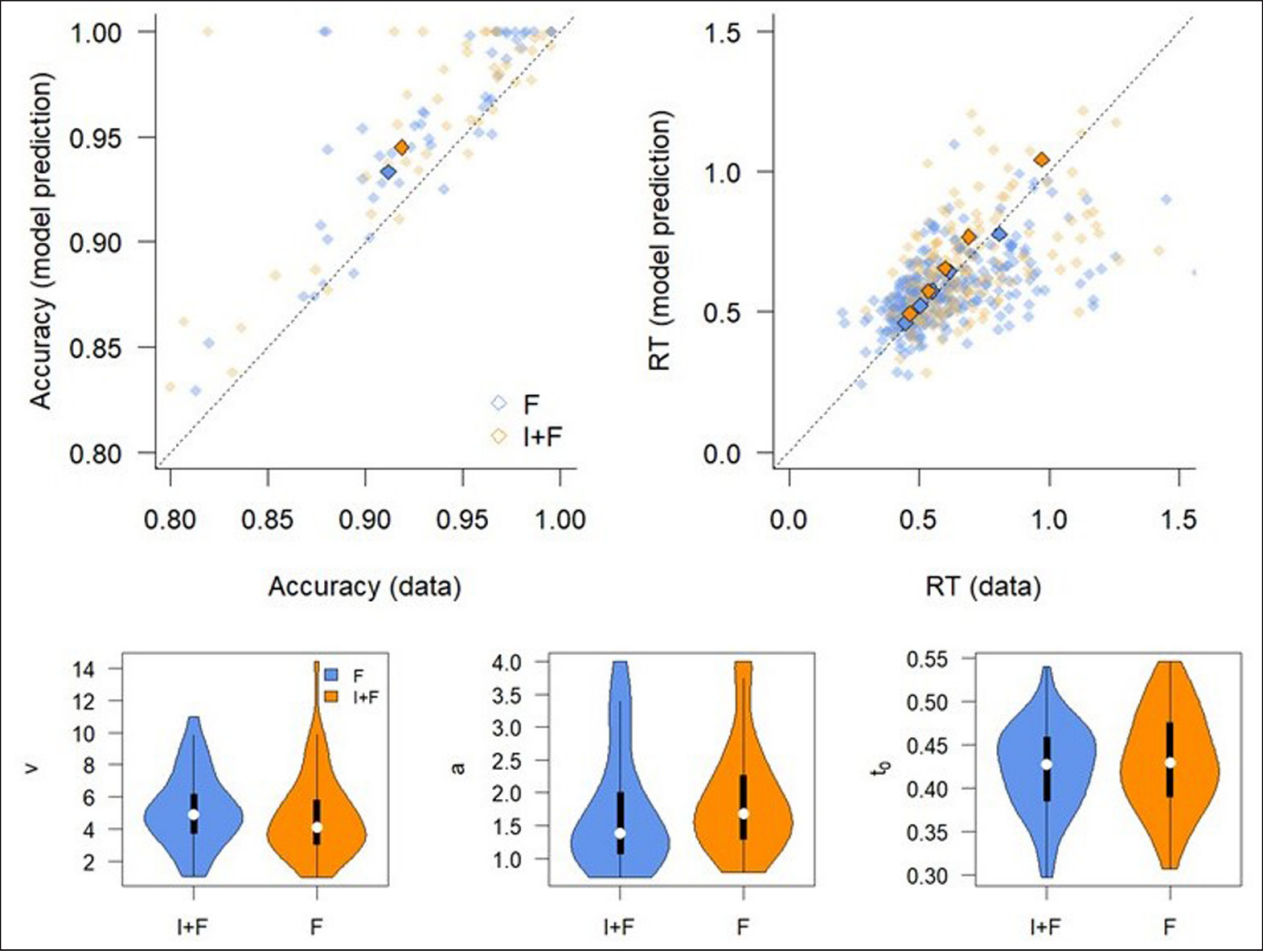
Fit of Diffusion Decision Model (Top panel) and model parameters (Bottom panel) reveal that there are no lasting effects of instruction. Top. QQ-plots of model fits. Individual data points refer to individuals, thick shapes indicate the mean of the 0.1, 0.3, 0.5, 0.7, and 0.9 quantile RTs. Bottom. Parameter estimates. Left: drift rate; Middle: boundary separation; Right: non-decision time. I+F: Instruction and feedback; F: feedback only.

We fit the Diffusion Decision Model to the last 15 repetitions of each SR mapping. Figure 9 (Top) shows that the model on average fits the data well, although the slight overestimation of the accuracy remains from Experiment 1. Figure 9 (bottom) shows that the DDM parameters do not differ between conditions (drift rate v: BF_01_ = 3.49; boundary separation a: BF_01_ = 2.55; non-decision time t_0_: BF_01_ = 3.47, all in support of the null hypothesis). Although this evidence is not overwhelming, it clearly goes against the alternative hypothesis that there are differences between conditions. When considered in combination with findings from Experiment 1, this seems convincing. In contrast to Experiment 1, participants now all reached asymptotic performance and again no long-lasting effects of instructing on the learning of SR mappings were observed.

## General Discussion

The present study aimed to understand the effect of instructions on behavior in a choice-reaction task. In particular, we addressed the question whether instructions have an effect during the automatic execution of the task (Chein & Schneider, 2012). Some researchers have argued that instructions have a lasting effect (Abrahamse et al., 2022; Bugg & Scullin, 2013; Pfeuffer et al., 2017; Popp et al., 2020; Wenke et al., 2009). Others have argued that instructions only have a temporary effect, that is superseded once an SR episode is formed in long-term memory (Schmidt et al., 2020). Addressing this question is not straightforward, because behavior changes over time, typically in a non-linear fashion (Anderson et al., 1999; Heathcote et al., 2000; Newell & Rosenbloom, 1981). Moreover, potential effects may manifest in multiple behavioral outcomes, such as response times or performance accuracy (Heitz, 2014; Van Maanen et al., 2019; Wickelgren, 1977). As a result, small effects in any of those behavioral measures may not be detectable.

To counter these issues, we took a two-step approach to analyze the data from our experiments. The first step was to focus on RT only, considering the non-linear learning behavior over repeated executions of the SR mappings. This analysis revealed that in both our experiments, instructing SR mappings has no effect on the final behavior. However, in Experiment 1, the parameter that represented the initial level of learning was increased, suggesting that instructions boost initial performance, essentially providing a head start for instructed SR mappings as compared to no-instructed SR mappings. We did not replicate this finding in Experiment 2. We speculate that this reflects the between-participants manipulation of the instructions, which contrasts with the within-participants manipulation in Experiment 1. That is, perhaps the presence of the Feedback-only condition boosts the effect of the Instructions in subsequent blocks. This interpretation is supported by a post-hoc analysis of the 8:1 Mapping individuals from Experiment 1. After splitting for block order, we found anecdotal evidence for an interaction effect in the *B* parameter estimates (BF_10_ = 1.15), such that the difference between the Instruction-and-Feedback and the Feedback-only conditions was slightly diminished for participants who started with those respective blocks. However, given the small sample size after splitting for block order, this result should be treated with caution.

The second step in our analysis was to focus on the final set of repetitions, which we considered to represent stable behavior during automatic task execution. To address potential speed-accuracy trade-offs, those data were analyzed using the DDM, a canonical decision-making process model. In Experiment 1, we found that for the simpler tasks with few SR mappings there was no evidence for a difference between the Instruction-and-Feedback and the Feedback-only condition, and in fact there was evidence against such a difference.

For the more complex SR mappings, the picture was a bit more nuanced. In Experiment 1, we observed evidence against a lasting effect of instruction for those individuals for whom it was clear that they reached asymptotic performance. However, some participants did not reach this level of performance yet, and consequently there was a difference between the Instruction-and-Feedback and the Feedback-only condition for those individuals. Exploratory regression analyses supported this interpretation. Participants for which we found evidence for a difference in asymptotic behavior in the first analysis – suggesting that learning was not finished yet – also offered more evidence for a difference between the two instruction conditions in the final set of repetitions. Moreover, a difference in the relevant parameter of the non-linear regression model were associated with a difference in relevant DDM parameters: Participants who had not fully automatized the task required more processing of the items in the feedback only blocks, yielding a lower drift rate. The items that were supported by instruction still benefited from this, resulting in faster processing and a higher drift rate.

The interpretation that any differences on the last set of repetitions in Experiment 1 stem from individuals who were still learning the task was supported by the results from Experiment 2. This experiment focused only on the hardest mapping task (8:1), but we extended the task to include 60 repetitions. This manipulation ensured that indeed all participants reached the stable behavior. Subsequent DDM analyses showed that there is no evidence for a lasting effect of instruction.

Although the results of Experiment 1 indicated the presence of general differences between the different mapping conditions, we did not consider these closely, as they possibly reflected confounded effects. The set-size manipulation we used also affected the block structure of the tasks in Experiment 1. This could have induced differences in fatigue, the learning of the task contingencies, and the spacing of the items. These factors are known to influence behavior, especially in learning experiments (e.g., Couto et al., in press; Van Rijn et al., 2009). Consequently, behavior in the last set of repetitions between mapping conditions may not be completely comparable, since those repetitions are earlier in the task but less spaced for the simpler mappings than the more complex mappings. However, these confounding effects did not jeopardize our conclusions about instructions, because the contrast between the Instruction-and-Feedback and the Feedback-only conditions was considered within each mapping block.

Taken together, we found some support for the conclusion that the beneficial effect of instructions does not reach the later phases of learning, such as the controlled- and automatic-execution phases proposed by Chein and Schneider (2012). This finding is in line with the computational model of Schmidt and colleagues (2020) in which it is assumed that instructions lead to initial SR traces that are quickly replaced by newer SR traces that are formed on the basis of actual practice (see also Cohen-Kdoshay & Meiran, 2007, 2009 for a similar idea). Based on the current findings, we can add to this model that instructions have no effect on the parameters underlying further practice of skill. In particular, whereas instructions may offer a head start when learning a new task, instructions do not seem to change the final outcome of the learning process.

Because our primary hypothesis entailed the absence of long-lasting effects of instruction, we mostly relied on Bayesian statistics that allow us to draw conclusions about the absence of an effect (Morey & Rouder, 2012; Rouder et al., 2012). Bayesian tests reveal uncertainty about the test outcome that, for example, would occur when the experiment would be underpowered. In that scenario, we would have found Bayes Factors close to 1. Although for some specific analyses we indeed report inconclusive results, the main analyses report substantial evidence in favor of the null hypothesis that there are no long-lasting effects of instruction. For this reason, the possibility of a lack of power can be excluded.

We must be cautious not to overgeneralize the current findings. Indeed, a lay person’s idea of “instruction” might not readily map onto the way we operationalized it, and other findings do suggest the presence of long-lasting effects of instructions, albeit by using a different behavioral proxy. The conclusions of Abrahamse et al., (2022) are based on the observation of longer lasting automatic response activations of instructed SR mappings (see also Pfeuffer et al., 2017). Such automatic activations are observed in situations in which the execution of instructed mappings is delayed or absent. As such, these mappings can be actively maintained or can linger on in memory. In contrast, in the current setting the instructed SR mappings were immediately executed and thus probably overwritten. Delaying the execution of instructions may thus have increased the longevity of SR traces in memory.

At the same time, other studies do show long-lasting effects of instructions even when instruction implementation is not delayed. For instance, Popp et al., (2020) observed that the initial chunking of instructions in a discrete sequence production task can influence performance even after several days of practice. One possible difference with the current experiment, is that such a task is more difficult and multilayered. Reiterating the Introduction, the translation from instruction to action is more complicated for a complex task. Possibly, the task model needs to include an hierarchical structure and information chunks need to be created (Bhandari & Duncan, 2014; Duncan et al., 1996, 2008; Verbruggen et al., 2018). It is likely that such a task model may lead to a more stable advantage compared to a situation in which a relatively complex task needs to be learnt on the basis of trial-and-error alone.

To conclude, we did not observe long-lasting effects of instructions and adhere to the view that for simple tasks the beneficial effect of instructions is limited. However, we do not exclude that for more complex tasks instructions may be more long-lasting. At the same time, we have demonstrated that the formalization of learning by means of exponential curves can be accommodated to investigate more complex instances of learning.

## Data accessibility statement

The data and analysis scripts of both experiments are available on OSF: https://osf.io/q8sa2/.

## Footnotes

The standard formulation of DDM does not directly assume between-trial variability in boundary separation, but this can be approximated by between-trial variability in the starting point of accumulation, which here we assume is a/2.

## Appendix A

Because we did not observe any effect of the presentation of object names on behavior in the 4:1 mapping condition, we collapsed those individuals from here on (BF_RT_ = 5.49; BF_acc_ = 5.09; both in favor of the null hypothesis).

Figure 2 in the main text suggests that after 30 repetitions of a SR mapping, behavior seems to stabilize, independent of instruction. To test this null hypothesis, we performed Bayesian hypothesis tests to compute the probability that the null hypothesis is true (Morey & Rouder, 2012; Rouder et al., 2012). A Bayesian linear model where RT was only predicted by Mapping was 72 times more likely than a model that additionally included Instruction and Repetition as factors and 26996 times more likely than an intercept-only model. This shows that when focusing on the last 10 repetitions, there is only an effect of Mapping on RT, and not of Instruction, nor of Repetition. In terms of response accuracy, a Bayesian linear model including only Mapping is 2916 times more likely than a model including Interaction and Repetition as well. However, due to a ceiling effect on accuracy the intercept-only more is 50 times more likely than the Mapping only model.
